# The MamaMiso study of self-administered misoprostol to prevent bleeding after childbirth in rural Uganda: a community-based, placebo-controlled randomised trial

**DOI:** 10.1186/s12884-015-0650-9

**Published:** 2015-09-14

**Authors:** Andrew D. Weeks, James Ditai, Sam Ononge, Brian Faragher, Laura J. Frye, Jill Durocher, Florence M. Mirembe, Josaphat Byamugisha, Beverly Winikoff, Zarko Alfirevic

**Affiliations:** Sanyu Research Unit, Department of Women’s and Children’s Health, University of Liverpool, Liverpool Women’s Hospital, Crown Street, Liverpool, L8 7SS UK; Sanyu Africa Research Institute (SAfRI), Mbale Regional Referral Hospital; and Sanyu Research Unit, Department of Women’s and Children’s Health, University of Liverpool, Liverpool Women’s Hospital, Crown Street, Liverpool, L8 7SS UK; Department of Obstetrics and Gynaecology, Makerere University College of Health Science, P.O Box 7072, Kampala, Uganda; Liverpool School of Tropical Medicine, Pembroke Place, Liverpool, L3 5QA UK; Gynuity Health Projects, 15 East 26th Street, Suite 801, New York, NY 10010 USA; Department of Women’s and Children’s Health, University of Liverpool, Liverpool Women’s Hospital, Crown Street, Liverpool, L8 7SS UK

## Abstract

**Background:**

600 mcg of oral misoprostol reduces the incidence of postpartum haemorrhage (PPH), but in previous research this medication has been administered by health workers. It is unclear whether it is also safe and effective when self-administered by women.

**Methods:**

This placebo-controlled, double-blind randomised trial enrolled consenting women of at least 34 weeks gestation, recruited over a 2-month period in Mbale District, Eastern Uganda. Participants had their haemoglobin measured antenatally and were given either 600mcg misoprostol or placebo to take home and use immediately after birth in the event of delivery at home. The primary clinical outcome was the incidence of fall in haemoglobin of over 20 % in home births followed-up within 5 days.

**Results:**

748 women were randomised to either misoprostol (374) or placebo (374). Of those enrolled, 57 % delivered at a health facility and 43 % delivered at home. 82 % of all medicine packs were retrieved at postnatal follow-up and 97 % of women delivering at home reported self-administration of the medicine. Two women in the misoprostol group took the study medication antenatally without adverse effects. There was no significant difference between the study groups in the drop of maternal haemoglobin by >20 % (misoprostol 9.4 % vs placebo 7.5 %, risk ratio 1.11, 95 % confidence interval 0.717 to 1.719). There was significantly more fever and shivering in the misoprostol group, but women found the medication highly acceptable.

**Conclusions:**

This study has shown that antenatally distributed, self-administered misoprostol can be appropriately taken by study participants. The rarity of the primary outcome means that a very large sample size would be required to demonstrate clinical effectiveness.

**Trial registration:**

This study was registered with the ISRCTN Register (ISRCTN70408620).

## Background

Postpartum haemorrhage (PPH) is one of the most common causes of maternal mortality worldwide [1]. It is responsible for around 30 % of maternal deaths, equivalent to 86,000 deaths per year annually or 10 deaths every hour. An important strategy in the prevention of postpartum bleeding is the prophylactic use of uterotonic drugs. The most commonly used drug is oxytocin, but its public health impact is limited by the need for parenteral administration by a health worker and a limited shelf life if not refrigerated. Misoprostol has been recognized as an option for preventing PPH in home births, as it is economical, heat stable, has a long shelf life, and can be taken orally. A meta-analysis of randomized studies comparing prophylactic use of misoprostol vs. nothing in community births assisted by traditional birth attendants (TBAs) or midwives shows a reduction in severe PPH rate [2]. Population coverage can be increased by the antenatal provision of misoprostol to women for self-administration at the time of delivery [3]. This strategy has been implemented by several programmes, which have reported reductions in PPH [4, 5].

Although misoprostol appears to be safe and effective in the community when administered by trained health workers [6–9], there have been concerns expressed about the risks of self-administration [10]. Furthermore, there is no evidence from randomized controlled trials (RCTs) on the benefits or risks of a strategy of advance distribution for self-administration [11]. To realize the benefits of self-administration, women need to ensure they have the medicine accessible at the time of delivery, and they need to swallow the medicine at the appropriate time. Concerns regarding this strategy include the possibility of discouraging facility deliveries, delaying treatment seeking, producing unmanageable side effects, and endangering a woman’s health through mis-timed administration. If these problems occurred, they could negate the benefits of widespread access to an effective uterotonic agent. If so, then the efficacy shown in clinical trials in which health workers administer the misoprostol may not translate into effectiveness in practice following its advance distribution for self-administration.

A randomised trial was therefore proposed in order to address the unanswered questions and concerns about the safety, effectiveness and feasibility of distributing misoprostol tablets in advance to women for self-use in home births to prevent PPH.

## Methods

In June 2008, misoprostol was approved by the Ministry of Health for the prevention and treatment of PPH in Uganda. Use by women for self-administration, however, is not currently practiced or promoted in Uganda. Furthermore, the policy of the Ministry has been to increase institutional births and not to encourage home births assisted by TBAs or village health workers.

### Study population

The study was conducted in Mbale district, Eastern Uganda, with recruitment in Mbale Regional Referral Hospital and 3 large health centres (Busiu, Lwangoli and Siira). The overall antenatal attendance is 95 % but only 57 % of women deliver in a health facility [12].

A list of 200 eligible villages that were served by the participating health facilities and had active village health teams (VHTs) was drawn up. All pregnant women at >34 weeks of gestation living in the recruitment villages were eligible to participate. This gestation was chosen so as to recruit women as close as possible to the date of birth whilst accepting an underlying rate of preterm birth and the inaccuracies of dating based on last menstrual period alone. Women with a known allergy to misoprostol or other prostaglandin, or under 18 years old (unless she was an emancipated minor) were excluded. Women at risk of experiencing a delivery complication were not excluded.

### Study objectives

The study objective was to pilot the study design for a possible larger study, including the logistics of community antenatal distribution of misoprostol and the feasibility of following-up participants. We also sought to determine the recruitment rate and the incidence of adverse events to provide data for sample size determination. No formal sample size calculation was conducted. Instead, the recruitment was time-limited to 2 months. During the recruitment period, the investigators together with the chairs of the Trial Steering Committee and Data Monitoring Committee (DMC) agreed on an *a priori* data analysis plan to examine the clinical effectiveness and safety of self-administered misoprostol, as well as compliance and feasibility around antenatal distribution and self-use of the medicine. This plan was developed in order to reduce the risk of bias inherent in *post hoc* changes, and forms the basis for the data analysis in this paper.

### Consent procedures

Ethical approval was obtained from the Liverpool School of Tropical Medicine Research and Ethics Committee (ref: 11 · 62) and the Mbale Referral Hospital Institutional Review Committee (ref: REIRC 010/2011). Posters were placed in the antenatal clinics and formal announcements made at the daily antenatal clinic to inform women about the study. Eligible women attending antenatal clinic received an information sheet before signing informed consent (or indicating consent with a mark as necessary). All study documents were available in English and the 3 local languages (Lugisu, Lugwere and Ateso) and were read aloud to women unable to read.

### Study procedures

**Fig. 1:**
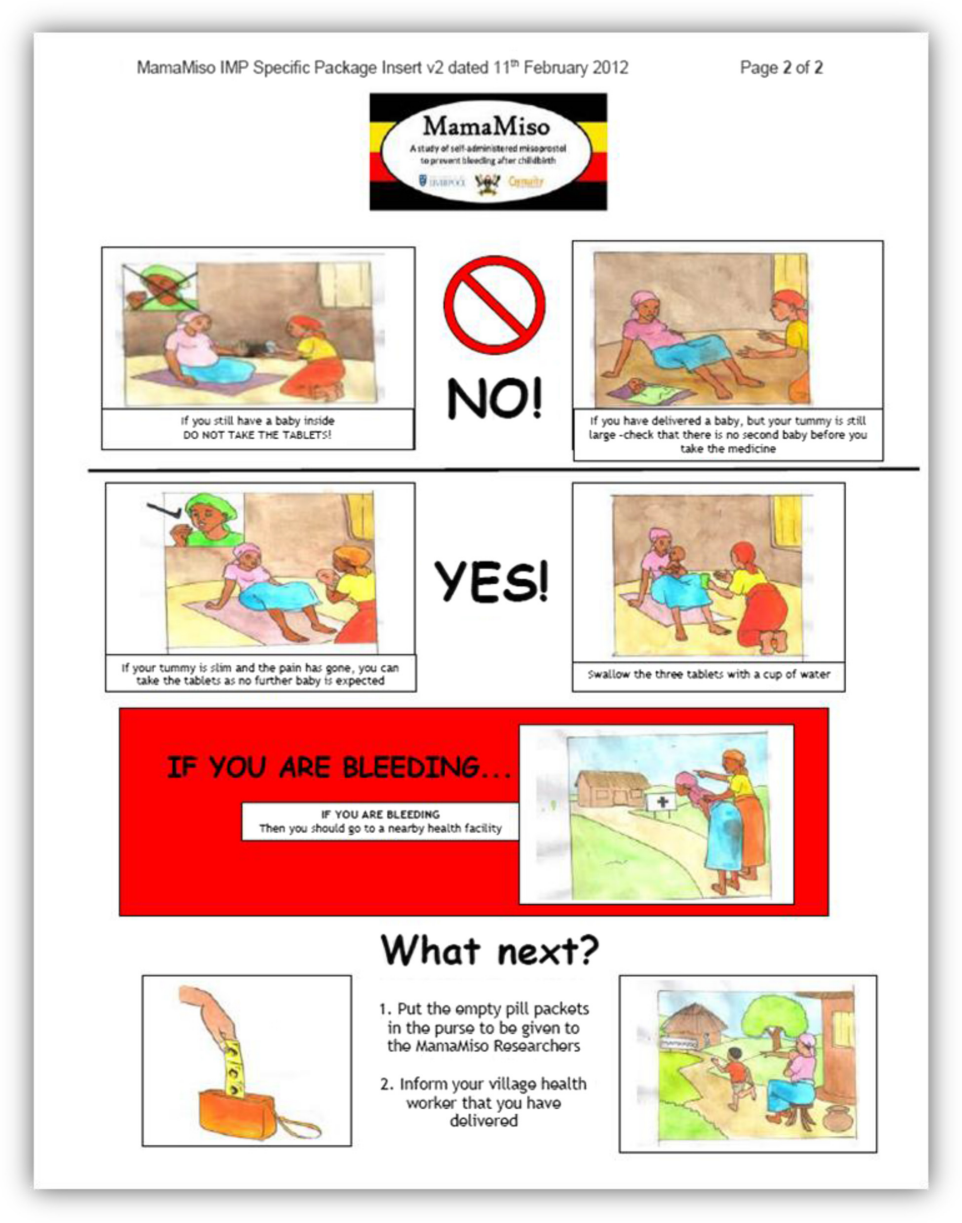
Pictorial instructions on how to take the tablets given to women antenatally along with the medicine packet (the first page had detailed instructions on study procedures)

Women who agreed to participate had an initial capillary haemoglobin measurement (Hemocue**®**, Angelholm, Sweden) taken at the time of enrolment during their third trimester antenatal care (ANC) visit. At this time, participants were randomized and given a small purse with a string that could be hung around the neck containing a packet with 3 foil-packed tablets (misoprostol or placebo) along with an instruction sheet with both pictorial and written instructions on how to take the tablets (Fig. 1). The packets were consecutively numbered according to a computer generated blocked randomisation code and contained either misoprostol 600 mcg (three 200 mcg tablets; GyMiso®) or identical placebo prepared by Linepharma (Paris, France). Both women and providers were blinded to study group assignment.

Women were taught to self-administer the medication orally, immediately after childbirth before the delivery of the placenta, and after confirming the absence of a twin, if they delivered at home. They were asked to retain the drug packaging following the birth, whether they took the medication (e.g. opened foil blisters) or not (e.g. sealed, unused packet). At recruitment, women were also educated about PPH and the importance of seeking care immediately if excessive bleeding is observed during a home delivery. The teaching materials developed and used by JHPIEGO in their community studies [4, 5] were the basis for those used in this study.

Women who went to hospital or health centres to deliver were advised not to take the tablets but to return the randomisation envelope to the health centre staff. The study team visited local health facilities each week to determine if any of the participants had delivered at that facility and to collect any unused/returned study medication.

After recruitment into the study, the participants continued to receive the standard antenatal care from the local antenatal clinic (including universal iron therapy, de-worming pills, tetanus toxoid and anti-malarial tablets). The study protocol did not include the provision of additional services beyond those already available at the clinic. All women were advised to deliver in a health facility as per national guidelines.

Following the recruitment of any rural woman, a VHT member in the participant’s village was informed and asked to keep in close contact with the study participant. Participants were also asked to remain in contact with the study team. Soon after delivery the VHT, clinic midwife or the woman herself were asked to contact the study team. The study team also contacted any women who had passed their estimated delivery date to identify those who had delivered. Researchers then visited the participant (ideally at 3–5 days after birth) either at home or in the health facility to assess the health of the mother and baby. A further capillary blood sample was taken to measure the change in haemoglobin level. Multiple other studies use this time for postnatal haemoglobin assessment [8, 9, 13, 14], as it is the lowest point for haemoglobin postnatally [15].

### Outcome measurement

Background and delivery characteristics, including whether the study medicine was self-administered and the timing of administration, were recorded for all participants. The primary clinical outcome was haemoglobin fall of over 20 %. Other haemoglobin outcomes included the mean change in haemoglobin and the rate of postnatal anaemia. Secondary clinical outcomes included the rate of poor maternal and fetal health, self-reported side effects, and safety (to include transfer to hospital, surgical intervention, blood transfusion and maternal death). At follow up, women were also asked to describe their perceived blood loss after birth, as well as respond to a series of questions that assessed the wellbeing of the mother and baby during the immediate postnatal period. This paper presents the main clinical and safety outcomes of the study, as well as participant compliance and the feasibility of self-administered misoprostol for the prevention of PPH among home births. Self-assessed blood loss and quality of life measures will be reported separately.

The analysis of Hb outcomes was a *per protocol* analysis confined to those who gave birth outside of an institution and who were followed up within 5 days. All other analyses included a ‘modified intention-to-treat’ group (analysing all women who were followed-up), and an analysis limited to those women followed up after a non-institutional birth. The data were analysed using SPSS statistics (version 21).

Any serious adverse events up to the time of follow-up were reported to the chair of the DMC and Gynuity Health Projects in accordance with the pharmacovigilance policy. The study was conducted according to CONSORT guidelines.

## Results

### Study participants

Overall, 748 women were recruited to the study over the two month period 23^rd^ May 2012 to 17^th^ July 2012. All eligible women consented to be in the study. The median (IQR) age of participants was 25 (21–30) years. Nearly 75 % of women were housewives; 93 % had completed primary education and 40 % had completed secondary education or higher. Review of the background characteristics shows that there were no differences between the study groups (Table 1), either as a whole, amongst those who had home births or for all women followed up (data not shown).

**Table 1:** Background characteristics for all women recruited into the study (*n* = 748)

	Misoprostol	Placebo
Number of women	374	374
Site of recruitment (*n*, %):		
Mbale Regional Referral Hospital	157 (42.0)	153 (40.9)
Busiu (level IV health centre)	119 (31.8)	119 (31.8)
Lwangoli (level III health centre)	56 (15.0)	54 (14.4)
Siira (level III health centre)	42 (11.2)	48 (12.8)
Age (mean, SD)	26.4 (6.2)	26.2 (6.4)
Primary occupation (*n*, %):		
Housewife	277 (74.1)	284 (75.9)
Employed	85 (22.7)	80 (21.4)
Unemployed / student	fc12 ( 3.2)	10 ( 2.7)
Highest level of education completed^†^ (*n*, %):		
No education	24 ( 6.4)	27 ( 7.2)
Primary	202 (54.2)	197 (52.7)
Secondary or higher	147 (39.3)	150 (40.1)
Estimated gestational age (mean, SD)	35.3 (1.4)	35.3 (1.5)
Nulliparous (*n*, %)	81 (21.7)	83 (22.2)
Previous CS deliveries (*n*, %)		
0	365 (97.6)	367 (98.1)
1	7 ( 1.9)	7 ( 1.9)^‡^
2	2 ( 0.5)	0
Haemoglobin (mean g/dl, SD, range))	11.2 (1.4) [6.9 – 14.7]	11.2 (1.4) [5.8 – 14.8]
Normal	207 (55.3 %)	214 (57.2 %)
Mild anaemia	136 (36.4 %)	127 (34.0 %)
Moderate anaemia	27 ( 7.2 %)	31 ( 8.3 %)
Severe anaemia*	3 ( 0.8 %)	2 ( 0.5 %)

† : not recorded for 1 participant in misoprostol group

‡: the number of previous CS was not recorded for one participant and a single CS was assumed

* : anaemia shown by WHO classification: normal (>11 g/dl), mild (9–10.99 g/dl), moderate (7–8.99 g/dl) and severe (<7 g/dl). Data is missing for one participant in the misoprostol arm

**Fig. 2:**
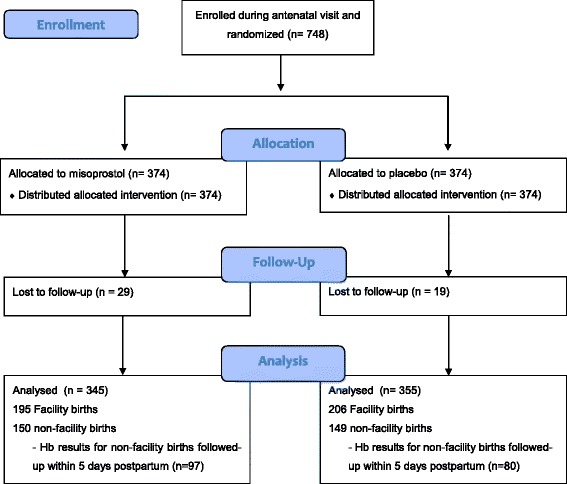
CONSORT trial profile

Follow-up data were obtained for 700 (93 · 2 %) of the women recruited (Fig. 2). The overall home birth rate was 43 % (Table 2). The median (IQR) time to follow up was 4 (3–17) days.

**Table 2:** Birthplace outcomes – all women followed up (*n* = 700)

	Misoprostol	Placebo	Relative risk (95 % CI)
Sample size	345	355	
Final place of delivery (*n*, %)			
Hospital	102 (29.6)	114 (32.1)	---
Government or private clinic	93 (27.0)	92 (25.9)	1.065 (0.871 : 1.302)
Home (own/family member)	125 (36.2)	120 (33.8)	1.076 (0.892 : 1.298)
House of friend or TBA	16 ( 4.6)	19 ( 5.4)	0.968 (0.657 : 1.462)
Other*	9 ( 2.6)	10 ( 2.8)	1.059 (0.668 : 1.678)
Number of babies delivered (*n*, %)			
1	338 (98.0)	347 (97.7)	---
2 or more	7 ( 2.0)	8 ( 2.3)^†^	1.002 (0.981 : 1.024)
Mode of delivery (*n*, %)			
Normal vaginal delivery	328 (95.1)	341 (96.1)	---
Caesarean section	16 ( 4.6)	14 ( 3.9)	1.088 (0.772 : 1.534)
Instrumental delivery	1 ( 0.3)	0	---
Person assisting with the birth**(*n*, %)			
Doctor and/or trained midwife	196 (56.8)	207 (58.3)	0.970 (0.836 : 1.125)
Traditional birth attendant	37 (10.7)	39 (11.0)	0.987 (0.782 : 1.245)
Family member and/or friend	106 (30.7)	103 (29.0)	1.041 (0.885 : 1.225)
No-one	12 ( 3.5)	13 ( 3.7)	0.974 (0.664 : 1.410)
Village Health team / Nursing assistant	3 ( 0.9)	4 ( 1.1)	0.886 (0.465 : 1.691)
Study medication taken (*n*, %)	204 (59.1)	193 (54.4)	1.088 (0.955 : 1.238)
If medication taken when did you take them? (*n*, %)			
Before labour started	1 ( 0.5)	0	---
During labour	1 ( 0.5)	0	---
Immediately after delivery (before placenta)	97 (47.5)	103 (53.4)	---
Immediately after delivery (after placenta)	102 (50.0)	87 (45.1)	1.113 (0.916 : 1.351)
1–24 hours after delivery	3 ( 1.5)	3 ( 1.6)	1.031 (0.457 : 2.324)

* : 14 mothers delivered en-route to hospital; 5 mothers delivered at the home of an unspecified health professional

** : more than one response possible so numbers do not total 100 %

† : two participants in the placebo arm recorded singleton births but stated verbally later that they delivered twins. They are both added to the multiple birth group in this table

### Medication use and safety

The study medication was reported having been taken by 290/299 = 97 · 0 % of mothers who gave birth at home and by 107/401 = 26 · 7 % of those who delivered in facilities (overall 56 · 7 %, Table 2). Medication packets (used and unused) were retrieved from most women who were followed up (575/700 = 82 · 1 %). The majority of women who took the study medication did so without assistance (74 · 3 %). Only 1 woman delivering at home did not have the medication with her at the time of the birth. In total, fifteen (2.1 %) women had multiple births; 5 women who delivered twins at home took the study medication correctly, after delivery of the second twin.

Among all women followed up, two women took the medication prior to the delivery of a singleton baby. Both had been randomised to misoprostol. One had visited a nearby TBA with ‘false labour pains’ and was instructed by the TBA to take the tablets immediately. She gave birth at a government clinic 6 days later. The other woman delivered at the home of a TBA and took the study medication during labour, one tablet before and two tablets after the delivery. Both had uncomplicated vaginal deliveries with no reported neonatal complications.

In the misoprostol arm, three women were hospitalised postnatally for postpartum haemorrhage; one of these women received a blood transfusion. All three women had facility births, did not take the study medication, and were subsequently discharged. There was 1 maternal death in a previously healthy parous woman, who had laboured at a health centre where there was no qualified midwife present. She had marked intrapartum blood loss and continued to bleed postnatally. She was not given any medication and was referred to Mbale Regional Referral Hospital, but died in transit. The study drugs (misoprostol) were not taken, but later retrieved from the caregiver. Lastly, one woman who was randomized to receive misoprostol but did not take the study medication was hospitalised postnatally following incision and drainage for a breast abscess.

In the placebo group, two women had postpartum haemorrhage (one of whom was transfused), 1 woman had a subtotal hysterectomy following a ruptured uterus, and 1 had an abdominal dehiscence following emergency caesarean section. All were facility births.

### Haemoglobin outcomes

Antenatal haemoglobin levels for all the women enrolled varied between 5 · 8 and 14 · 8 g/dl with a mean (s.d.) of 11 · 2 (1 · 4) g/dl, while postnatal values varied between 4 · 4 and 15 · 8 g/dl with a mean (s.d.) of 11 · 4 (1 · 6).

The main group of interest were the 177 women who delivered outside of a formal health facility and had haemoglobin outcomes assessed within 5 days of delivery. There was no significant difference between the groups for the primary outcome of a fall in haemoglobin >20 % (misoprostol 9 · 4 % vs placebo 7 · 5 %, relative risk 1 · 11, 95 % confidence interval 0 · 72 to 1 · 72; Table 3). The mean change in haemoglobin was similar in the two groups (−0 · 06 vs −0 · 09; mean difference 0 · 03, 95 % CI −0 · 47 to 0 · 53). Similar results were found for those women who had home births and were followed up at any point in time postnatally (data not shown).

**Table 3:** Outcomes from the births of all women who gave birth at home (or family/TBA home). Data on haemoglobin is taken from those for whom a follow-up assessment was completed within 5 days of delivery

Haemoglobin Measures (women seen within 5 days of delivery)			
	Misoprostol (*n* = 97)	Placebo (*n* = 80)	Relative risk *or* mean difference (95 % CI)
Days from birth to follow-up (mean, (SD))	3.6 (0.7)	3.5 (0.7)	0.07 (−0.14 : 0.28)
Fall in Hb >20 % (*n*, %)	9 ( 9.4)	6 ( 7.5)	1.110 (0.717 : 1.719)
Fall in Hb > 2 g/dl (*n*, %)	13 (13.4)	8 (10.0)	1.150 (0.798 : 1.657)
Postnatal Hb g/dl (mean (SD) [range])	11.1 (1.8) [5.2:15.3]	11.2 (1.4) [6.1:14.0]	−0.11 (−0.59 : 0.37)
Anaemia status* (*n*,%)			
Normal	51 (52.6)	46 (57.5)	---
Mild	36 (37.1)	28 (35.0)	1.070 (0.803 : 1.426)
Moderate	8 ( 8.2)	5 ( 6.2)	1.170 (0.732 : 1.872)
Severe	2 ( 2.1)	1 ( 1.2)	1.268 (0.557 : 2.885)
Fall in Hb g/dl (mean (SD) [range])	0.06 (1.82) [−5.5:5.2]	0.09 (1.50) [−3.5:4.0]	−0.03 (−0.53 : 0.47)
Maternal and Neonatal Outcomes (all home births)			
	Misoprostol (*n* = 150)	Placebo (*n* = 149)	Relative risk *or* mean difference (95 % CI)
Days from birth to follow-up (median (IQR))	4 (3 – 13)	5 (3 – 25)	---
Maternal complications (*n*, %)^†^			
Health professional consulted	2 ( 1.3)	3 ( 2.0)	0.795 (0.525 : 1.427)
Admitted to a health facility >24 hours**	1 ( 0.7)	1 ( 0.7)	0.997 (0.517 : 1.691)
Surgical procedure since delivery***	0	1 ( 0.7)	---
Retained placenta	3 ( 2.0)	2 ( 1.3)	1.200 (0.581 : 2.477)
Blood transfusion after delivery	0	0	---
Use of additional uterotonics (*n*, %)	3 (2.0)	4 (2.7)	0.745 (0.189 : 2.928)
Self-reported side effects (*n*, %)^†^			
Shivering/chills after birth	75 (51.0)	40 (27.8)	1.594 (1.277 : 1.991)
Fever after birth	40 (27.2)	21 (14.6)	1.410 (1.121 : 1.772)
Diarrhoea within 24 hours	4 ( 2.7)	1 ( 0.7)	1.600 (1.017 : 2.518)
Neonatal Death (*n*, %)	0	3 ( 2.0)	---
If ‘yes, when did the baby die? (*n*, %)			
Before birth	0	1 (33.3)	---
Within 24 hours of birth	0	0	
More than 24 hours after birth	0	2 (66.7)	

* Anaemia shown by WHO classification: normal (>11 g/dl), mild (9–10.99 g/dl), moderate (7.8.99 g/dl) and severe (<7 g/dl).

** Misoprostol: admitted with Hb <6 g/dl; placebo: admitted for drainage of breast abscess

*** Drainage of breast abscess

† Respondents could have more than one response. Data missing for 3 in the misoprostol group and 5 in the placebo group.

### Clinical outcomes

Clinical maternal complications were rare among the cohort delivering at home (*n* = 299). No woman had a postnatal blood transfusion and only one had a subsequent surgical procedure (incision of a breast abscess). Seven women were given other/additional uterotonics by their birth attendant at home (misoprostol arm *n* = 3; placebo arm *n* = 4). Two women reported that they sought higher level of care for postpartum bleeding. One had delivered en route to the hospital and had not taken the study medication. She experienced heavy bleeding and when she reached the facility she was hospitalized for 3 days due to severe anaemia (Hb level 6.0 g/dL). Another woman in the placebo arm reported that she had consulted a health professional after having continuous bleeding for several days after delivery; she was diagnosed and treated for a urinary tract infection. There was one stillbirth and 3 neonatal deaths, all in the placebo group. Shivering was significantly more common in the misoprostol group (51 % vs 28 %, RR 1 · 59, 95 % CI 1 · 28 to 1 · 99) as was fever (27 · 2 % vs 14 · 6 %, RR 1 · 41, 95 % CI 1 · 12 to 1 · 77). Satisfaction rates were high in both groups with the vast majority (96 · 7 %) stating that they were satisfied with the medication (misoprostol 96 · 5 % vs placebo 96 · 9 %) and an even larger proportion indicating that they would recommend the prophylaxis to a friend (98 · 3 % in both the misoprostol and placebo groups).

Approximately one-quarter of women who had facility births also took the study drug (106/401 = 26 %), mainly due to a lack of availability of oxytocin. We therefore conducted a post-hoc analysis of all women who took the medication irrespective of delivery location and timing of follow up (*N* = 397). The rate of a >20 % fall in haemoglobin was similar between the groups (misoprostol 4 · 5 % vs placebo 5 · 2 %, RR 0 · 92, 95 % CI 0 · 57 to 1 · 50).

## Discussion

This study allows evaluation of the risks and benefits of a strategy of antenatal distribution of 600 mcg oral misoprostol for self-administration along with collection of individual-level outcome data for women who took the medicine following a non-institutional delivery. Overall, postpartum follow-up in this study was achieved in 93 % of participants, with 61 % seen within 5 days of delivery. Participant compliance in taking the prophylactic regimen at home, as well as women’s satisfaction-levels, were high (rates exceeding 97 % for both of these feasibility measures). These results corroborate other documented experiences about the feasibility of offering misoprostol to women for self-use at the time of delivery in order to prevent postpartum haemorrhage [3].

The close follow-up in this study has allowed a robust assessment of how misoprostol may be used in the community in an advance distribution programme. Only 2 of the 700 women who were given medicine packets in their third trimester took the tablets early with a fetus still *in utero.* Both of these women delivered healthy babies despite being in the misoprostol arm. Similarly, a self-administration study in Liberia saw no adverse events in the three women who took misoprostol prior to delivery.[16] While misoprostol use before delivery has the potential for causing harm, the effect of that mistimed administration will depend on the clinical situation and the dose and route of administration [17]. The potential danger would be increased if the labour were obstructed already, if she has a previous caesarean section scar, or if the baby was in a transverse lie, but these are comparatively rare. With well-designed programmes and widespread training of providers, women, and families on the correct use of this medicine, mistimed administration can be greatly minimized. Importantly, in our study, most women took the study drug in the right way after the birth of the baby(ies). This supports population data from other larger studies reporting minimal incorrect use before delivery [3–5, 16]. However, programs should continue to monitor adverse events to confirm that complications from early administration are not occurring under any other circumstances not captured in this study.

While instructions were to take the medicine after the birth of the baby and before the expulsion of the placenta, nearly half of women reported taking the tablets after placental delivery. Similarly, Smith et al. showed nearly a third of women in a sample in Liberia administered the medicine late [16]. This might be due to a rapid placental expulsion, poor recall of precise timing of self-use, or difficulties achieving this timing in a home setting. However, there is no reason to believe that uterotonic administration after placental expulsion would have adverse effects, although it might delay the onset of action. Optimal timing of uterotonic administration is still a question warranting further research.

One of the strengths of the study is the variation that was captured with regard to self-administration of the medicine, as well as the different homebirth delivery scenarios. Nearly three-quarters of home deliveries were unattended by a provider: 65 % (194/299) were assisted by a family member/friend and 8 % (24/299) did not have anyone present. Only one-quarter were assisted by a TBA. Although the homebirth cohort is relatively small in this study, the ‘unsupervised’ delivery environment provides important insight on how the medicine may be used without negative consequence when scaled-up in programs. Importantly, the results point to the need for improved antenatal counselling for women. Additional community sensitization activities targeting women and their families may also be helpful to emphasize key instructions about when to take the medicine. However, these findings and lessons learnt may not be generalizable to contexts where the delivery environment is different or for strategies in which drug dissemination occurs in a different way.

Among homebirths in this study, large peripartum falls in hemoglobin were uncommon and life-threatening PPH rare. Whilst this is a welcome finding, it makes the conduct of efficacy trials very difficult. Indeed, a very large trial would be needed to demonstrate a significant reduction in PPH outcomes associated with a self-administration strategy. For example, a 25 % relative reduction in ‘fall in haemoglobin of over 20 %’ from 7 · 5 % to 5 · 6 % would require 7,120 home births to be followed up within 5 days (α = 0 · 05; β = 0 · 9). In a setting with an institutional birth rate of 40–50 %, this might require around 15,000–20,000 recruits, depending on the feasibility of conducting postpartum follow-up visits within 5 days. Our time-limited study was not designed to assess the clinical effectiveness of misoprostol (700 women has only 17 % power to detect a fall in Hb of over 20 % from 7.5 % to 5.6 %, α = 0.05), and we found no statistically significant difference between the groups.

## Conclusions

On the basis of the data from this and other studies, there are no reasons to prevent a policy of antenatal distribution of misoprostol for self-administration. This conclusion is based on three factors:

1. Since MamaMiso was conceived, a large placebo-controlled study has been published [8], and there has also been ongoing experience with antenatal distribution of misoprostol [3–5, 11, 18, 19]. Meta-analysis of randomised trials now shows that community misoprostol reduces postpartum haemorrhage [2].
2. The remaining issue has been whether the potential problems with self-administration (women not having the medication with them at the time of labour or taking it incorrectly) would negate the benefits of widespread access to misoprostol in home birth settings. In this study we found that participants did have the medication with them at the time of delivery and were able to take the misoprostol appropriately after birth (albeit with many delaying it until after the delivery of the placenta). These data, combined with that of Smith [3] are strongly suggestive that the efficacy seen when health workers administer misoprostol in the community will also be seen when women self-administer the drug themselves.
3. Rare adverse events are best detected through observational studies or reporting systems, but individual randomized trials are also important to pick up more common but less severe safety problems. The safety demonstrated in this study adds to the safety data from other large observational studies [3–5, 16, 18, 19].

In conclusion, this study has shown that antenatally distributed, self-administered misoprostol can be safely taken by study participants. With careful counselling and monitoring of adverse events, programmes may move beyond safety concerns and focus on other implementation issues such as ensuring that the right population of women is reached and optimizing community education activities.

## Abbreviations

ANC: Antenatal care
DMC: Data Monitoring Committee
IQR: Interquartile range
PPH: Postpartum hemorrhage
RCT: Randomized-controlled trial
TBA: Traditional birth attendant
VHT: Village Health Team
